# Cyclophilin A Protects Cardiomyocytes against Hypoxia/Reoxygenation-Induced Apoptosis via the AKT/Nox2 Pathway

**DOI:** 10.1155/2019/2717986

**Published:** 2019-04-28

**Authors:** Fuyu Cheng, Wei Yuan, Mengfei Cao, Rui Chen, Xiuli Wu, Jinchuan Yan

**Affiliations:** ^1^Department of Cardiology, Affiliated Hospital of Jiangsu University, Zhenjiang, Jiangsu Province, China; ^2^Department of Cardiology, Jurong Pepole's Hospital, Jurong, Jiangsu Province, China

## Abstract

Hypoxia/reoxygenation (H/R) accelerates the process of cardiomyocyte apoptosis during ischemia-reperfusion. Excessive reactive oxygen species (ROS) are a critical driver of oxidative stress injury. Cyclophilin A (CyPA) is a major ROS-induced factor in atherosclerosis. There is a positive feedback mechanism between CyPA and ROS, which enables the oxidative stress response to continue and expand. However, it is unclear whether this positive feedback mechanism exists in cardiomyocytes. Through western blotting and flow cytometric assays and TUNEL assay, we found that CyPA inhibited the apoptosis of H9c2 cardiomyocytes under H/R conditions. By dihydroethidium (DHE) staining and electron spin resonance (ESR) assays, we demonstrated that CyPA reduced ROS production and suppressed O_2_^−^ production dependent on reduced nicotinamide adenine dinucleotide phosphate (NADPH) oxidase. By western blotting, we showed that CyPA inhibited the expression of NADPH oxidase 2 (Nox2) protein by the AKT pathway. Through confocal microscopy assay, we found that CyPA reduced the expression of Nox2 membrane-bound subunits. The current study shows that a positive feedback mechanism does not exist in H9c2 cardiomyoblasts. CyPA protects H9c2 cardiomyoblasts against H/R-induced apoptosis via the AKT/Nox2 pathway. This could be a potential target for ischemia-reperfusion injury therapy.

## 1. Introduction

In ischemia-reperfusion injury, the main cause of injury to the tissue is not the ischemia itself; the injury occurs after the recovery of the blood supply and is caused at least in part by excessive reactive oxygen species (ROS) attacking the cells in the tissue that regains its blood supply. Cyclophilin A (CyPA) is involved in the process of ischemia-reperfusion in tissues and exhibits different effects in a variety of cells. CyPA is a highly conserved protein in cells, which belongs to the cyclophilin family [1]. CyPA was the first member of the cyclophilin family to be discovered and is mainly located in the cytoplasm. It is widely distributed in almost all tissues [2, 3]. CyPA is classified into a variety of proteins termed foldases due to its enzymatic properties, its role in protein folding, and its cellular localization [4]. It is necessary for protein folding because of its enzymatic peptidyl prolyl cis-/trans-isomerase (PPIase) activity [5]. In addition to its role in protein folding, CyPA has been demonstrated to have a variety of functions, such as intracellular protein trafficking [6, 7], mitochondrial function [8, 9], maintenance of multiprotein complex stability [6], and pre-mRNA processing [10].

Although CyPA was once considered to be an intracellular protein, in recent years, it has been found that CyPA can be secreted outside the cell under conditions of inflammatory stimuli and oxidative stress [11, 12]. Recent studies have indicated that CyPA plays an irreplaceable role in the transport of NADPH oxidase (Nox) enzymes such as p47phox [13]. Recent studies have shown that Nox enzymes play a key role in tissues and organs during I/R [14, 15]. The Noxes are also known to cause vascular smooth muscle cell (VSMC) proliferation and vascular disease development [16]. In VSMCs, CyPA and Nox enzymes synergistically amplify ROS formation due to the activation of other oxidase systems by ROS produced by Nox enzymes [11], resulting in increased oxidative stress [17]. Although little is known about the role of CyPA in cardiomyocytes stimulated by hypoxia/reoxygenation (H/R), studies have suggested that the antioxidant activity of CyPA protects cancer cells against cell death under hypoxic conditions [18, 19]. The research of Boulos' team also suggests that extracellular CyPA can increase neuronal tolerance to oxidative stress [20]. Although the role of CyPA in cardiomyocyte apoptosis stimulated by H/R is still unclear, several reports have shown that CyPA is released from cardiomyocytes to cope with H/R, possibly protecting cardiomyocytes against oxidative stress-induced apoptosis [21]. All these reports indicate that CyPA may be critical for the antioxidant capacity of cardiomyocytes. In this study, we show that CyPA protects H9c2 cells against H/R-induced apoptosis, at least in part due to inhibition of NADPH oxidase activity.

## 2. Materials and Methods

### 2.1. Cell Culture and Hypoxia/Reoxygenation

The rat myocardial cell line H9c2 was purchased from the Cell Bank of the Shanghai Institutes for Biological Sciences, Chinese Academy of Sciences. H9c2 cardiomyocytes were grown in DMEM (Gibco, NY, USA) supplemented with 10% fetal bovine serum (FBS; Wisent, Montreal, Canada), 100 units/mL penicillin, and 100 units/mL streptomycin at 37°C with 5% CO_2_ in a humidified atmosphere. H/R conditions were produced by placing cells in a H/R chamber (1% O_2_, 94% N_2_, and 5% CO_2_) for a specific period of time.

CyPA preparation: CyPA (Abcam, ab86219) was dissolved in DMEM for the treatment of cells.

GSK690693 preparation: GSK690693 (MCE, HY-10249) is a novel Akt kinase inhibitor. GSK690693 was dissolved in dimethyl sulfoxide (DMSO) at a concentration of 10 *μ*M before use.

GSK2795039 preparation: GSK2795039 (MCE, HY-18950) is a NADPH oxidase 2 inhibitor. GSK2795039 was dissolved in DMSO at a concentration of 10 *μ*M before cell treatment.

### 2.2. Western Blot Analysis

Protein expression levels of BAX, Bcl-2, caspase 3, Nox1, Nox2, Nox4, AKT, p-AKT-Ser473, and *β*-actin were analyzed by western blotting as described above [22]. In brief, H9c2 cells were washed twice with phosphate-buffered saline (PBS) and lysed in lysis buffer for 30 min at 4°C. Cell lysates were centrifuged at 12,000 rpm for 15 min at 4°C. The protein concentration was measured with the BCA protein assay reagent kit (Thermo Fisher, 23227). After boiling for 5 min at 95°C in a 5× loading buffer, an equal amount of protein was separated by 12% SDS-polyacrylamide gel electrophoresis at 70 V for 30 min and 100 V for 1 h. Then protein was transferred to polyvinylidene fluoride membranes (0.45 *μ*m, Millipore Co. Ltd.) at 350 mA for 1 h. After blocking with 5% nonfat milk in Tris-buffered saline with Tween 20 for 1 h at room temperature and then washing three times with TBST, the membranes were incubated with primary antibodies against BAX (1 : 1000, Cell Signaling Technology), Bcl-2 (1 : 1000, Abcam), caspase-3 (1 : 1000, Cell Signaling Technology), AKT (1 : 1000, Cell Signaling Technology), p-AKT-Ser473 (1 : 2000, Cell Signaling Technology), Nox1 (1 : 1000, Abcam), Nox4 (1 : 1000, Abcam), and Nox2 (1 : 5000, Abcam) overnight at 4°C. *β*-Actin (1 : 1000, Cell Signaling Technology) was used as the control protein. The next day, the membranes were washed three times with TBST and incubated with an HRP-conjugated secondary antibody (1 : 1000, Cell Signaling Technology) for 1 h at room temperature. Bound antibody was detected with an Amersham Imager 600, and densitometric analysis of the images was performed using the ImageJ image processing program.

### 2.3. Flow Cytometric Assays

The FITC Annexin V Apoptosis Detection Kit I (BD Pharmingen, 556547) was used to detect apoptosis of H9c2 cells according to the manufacturer's instructions. H9c2 cells were trypsinized and then centrifuged at 1000 rpm for 5 min at room temperature. The cells were washed twice in cold (4°C) PBS, centrifuged again, and then resuspended in 300 *μ*L of 1× binding buffer. The resuspended cells were incubated with 5 *μ*L of Annexin V-FITC and 5 *μ*L of propidium iodide (PI) staining solution for 15 min without light at room temperature. After adding 200 *μ*L of 1× binding buffer, the cells were detected by flow cytometry on a FACS Calibur (BD Biosciences) within 1 h. The data were analyzed by FlowJo 7.6 software. The percentage of stained cells to total cells was computed as the ratio of apoptotic cells.

### 2.4. TUNEL Assay

Apoptosis in H9c2 cells was detected by using a high-sensitive one-step terminal deoxyribonucleotide transferase dUTP nick end labelling (TUNEL) apoptosis assay kit (Beyotime Institute of Biotechnology, Jiangsu, China) according to the manufacturer's instructions. In brief, H9c2 cells were fixed with 4% paraformaldehyde for 30 min. After washing with PBS, using 0.3% Triton X-100, H9c2 cells were permeabilized. Then they were incubated with 50 *μ*L TUNEL reaction fluid in a humid environment at 37°C for 1 h. And after washing twice with PBS, H9c2 cells were incubated with 4′, 6-diamidino-2-phenylindole (DAPI) to stain nuclei. Finally, H9c2 cells were observed under fluorescence microscope.

### 2.5. DHE Staining

Total O_2_^−^ production by H9c2 cells was measured by using dihydroethidium (DHE) (Sigma, CAS 104821-25-2). DHE is oxidized by intracellular O_2_^−^ and then it combines with the chromosomal DNA in the nucleus to generate red fluorescence. Treated H9c2 cells were washed twice with PBS. The washed cells were incubated with 20 *μ*M DHE diluted in 2 mL serum-free DMEM without light at 37°C. H9c2 cells were washed three times again with 2 mL PBS after 30 min incubation and then fluorescence microscopy was used to observe the red fluorescent images. ImageJ software was used to analyze the fluorescence intensity. The ratio of fluorescence intensity to the basal level was quantified and regarded as the cellular O_2_^−^ level.

### 2.6. ESR Assays

In order to detect the O_2_^−^ production which is dependent on Nox, we mixed 10 *μ*L of the protein samples collected from the H9c2 cells with 80 *μ*L of a solution made of Krebs-HEPES buffer which also contained diethyldithiocarbamate (5 *μ*M, Sigma) and deferoxamine (25 *μ*M, Sigma) and followed this by the addition of the cell-permeable spin probe 1-hydroxy-3-methoxycarbonyl-2,2,5,5-tetramethylpyrrolidine (CMH; ENZO, Alexis Corporation) to a concentration of 1 mM and the substrate NADPH (Beyotime Biotechnology) to a concentration of 20 *μ*M, with or without manganese-dependent superoxide dismutase (SOD, 8000 U/mL; Beyotime Biotechnology). An electron spin resonance (ESR) spectrometer (Bruker, Germany) was used to analyze O_2_^−^ production in the protein samples. In the homogenates, the SOD-inhibitable fraction of the signal showed the total O_2_^−^ production, and the results were indicated as fold changes compared with controls.

### 2.7. Confocal Microscopy Assay

A nuclear location of NOX2 in H9c2 cells was observed by confocal microscopy. In brief, H9c2 cells were fixed with 4% paraformaldehyde for 10 min. And after washing twice with PBS, using 0.5% Triton X-100, H9c2 cells were permeabilized. After washing twice again, using 1% Bull Serum Albumin (BSA) to block cells for 30 min at room temperature and then washing three times with PBS, the cells were incubated with primary antibodies against Nox2 (1 : 5000, Abcam) overnight at 4°C. The next day, the cells were washed three times with PBS and incubated with Goat Anti-Rabbit IgG (H&L) Alexa Fluor 594 secondary antibody (1 : 200, Abcam) for 2 h at room temperature. After washing twice with PBS, H9c2 cells were incubated with 4′, 6-diamidino-2-phenylindole (DAPI) to stain nuclei for 30 min without light. Finally, H9c2 cells were observed under confocal microscopy.

### 2.8. Statistical Analysis

Data are presented as the mean ± SE. The significance of the differences between two groups was examined using Student's *t*-test followed by Duncan's multiple-range test. Values of *p* < 0.05 were considered statistically significant.

## 3. Results and Discussion

### 3.1. CyPA Inhibits Apoptosis of H9c2 Cells under Hypoxia/Reoxygenation Conditions

**Figure 1:**
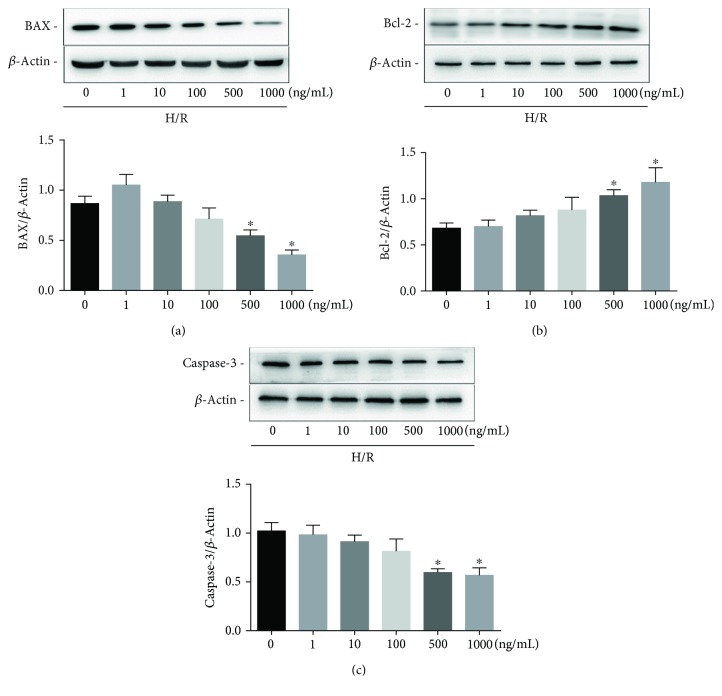
Expression of apoptosis-associated proteins induced by hypoxia/reoxygenation at different concentrations of CyPA. H9c2 cells were incubated with different concentrations of CyPA (0, 1, 10, 100, 500, and 1000 ng/mL) for 18 h of hypoxia and 4 h of reoxygenation. The protein expression levels of BAX, Bcl-2, and caspase-3 are revealed in the representative western blot gel files and summarized data (a–c). ^∗^*p* < 0.05 vs. the control group (*n* = 4).

**Figure 2:**
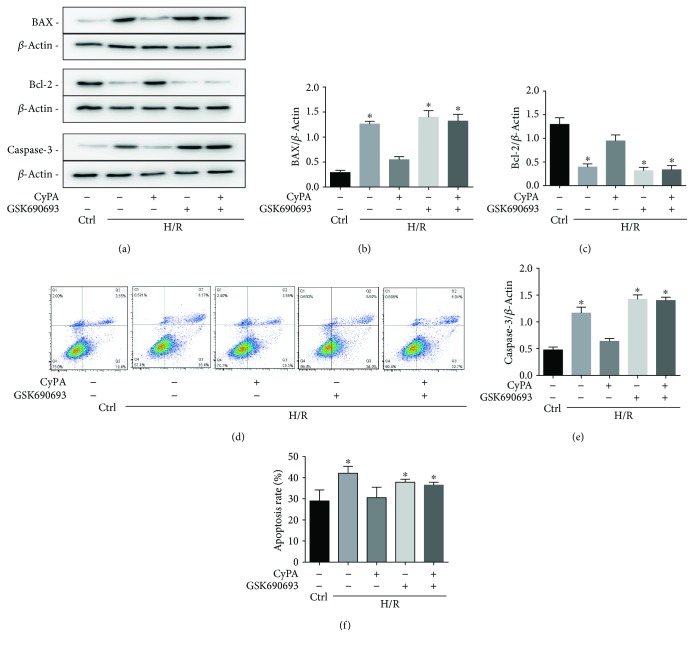
CyPA inhibits apoptosis of H9c2 cells under hypoxia/reoxygenation conditions. H9c2 cells were divided into five groups, control (cultured for 22 h under normoxic conditions), H/R (cultured for 18 h of hypoxia followed by 4 h of reoxygenation), H/R+CyPA (1000 ng/mL), H/R+GSK690693 (10 *μ*M), and H/R+CyPA (1000 ng/mL)+GSK690693 (10 *μ*M). The protein expression levels of BAX, Bcl-2, and caspase-3 are revealed in the representative western blot gel files and summarized data (a–c, e). The apoptotic populations of H9c2 cells sorted by flow cytometry after double staining with FITC-Annexin V and propidium iodide (PI) are revealed in representative plots (d) and summarized data (f).^∗^*p* < 0.05 vs. the H/R+CyPA group (*n* = 4).

In a variety of cell types, BAX and Bcl-2 can control proapoptotic and antiapoptotic intracellular signals which are very important in programmed cell death. In addition, the activation of caspase-3 also mediates apoptosis. Our preliminary experiments showed that H9c2 cells underwent significant apoptosis after 18 h of hypoxia and 4 h of reoxygenation. In these cells, we detected a large amount of ROS by DHE staining (not shown). We determined the concentration of CyPA needed for intervention and the effect of CyPA on H9c2 cells under H/R. On western blot analysis, we found that the most remarkable changes of BAX, Bcl-2, and caspase-3 occurred in response to 1000 ng/mL of CyPA (Figures 1(a)–1(c)). CyPA remarkably reduced the protein levels of BAX and caspase-3 (Figures 2(a), 2(b), and 2(e)). In contrast, expression of Bcl-2 was significantly increased in response to CyPA (Figures 2(a) and 2(c)). However, treatment with GSK690693 (a pan-Akt inhibitor targeting Akt1/2/3) prevented the effects of CyPA, and the expression levels of all apoptotic proteins in the H/R+CyPA+GSK690693 group were almost the same as those in the H/R+GSK690693 group (Figures 2(a)–2(c) and 2(e)). Annexin V-FITC/PI double staining was further used for flow cytometric assays to investigate the roles of CyPA in opposing apoptosis induced by hypoxia/reoxygenation. The results indicated that the percentage of H9c2 cells which bound Annexin V-FITC increased from 22.95% in the control group to 41.97% in the H/R group, while CyPA intervention significantly inhibited apoptosis, resulting in only 26.89% apoptotic cells. However, the percentage of cells in apoptosis increased to 38.74% in the H/R+CyPA+GSK690693 group (Figures 2(d) and 2(f)). And the percentage of cells in apoptosis is 39.92% in the H/R+GSK690693 group (Figures 2(d) and 2(f)).

**Figure 3:**
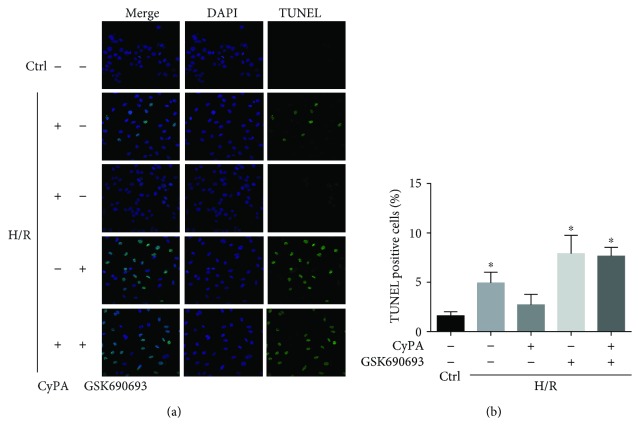
Representative fluorescent images of TUNEL assay and summarized data showing the TUNEL cells. Photographs were at ×400 magnification. ^∗^*p* < 0.05 vs. the H/R+CyPA group (*n* = 3).

We also further verified this result with the TUNEL kit. Normal cells hardly showed green fluorescence; TUNEL cells increased significantly in the H/R group and this situation improved after CyPA stimulation. But TUNEL cells increased again in the H/R+GSK690693 group and the H/R+CyPA+GSK690693 group (Figure 3). These data consistently demonstrated the antiapoptotic role of CyPA in H9c2 cells under H/R conditions and showed that this may occur by activating the AKT pathway.

### 3.2. CyPA Decreased O_2_^−^ Production under Hypoxia/Reoxygenation Conditions

**Figure 4:**
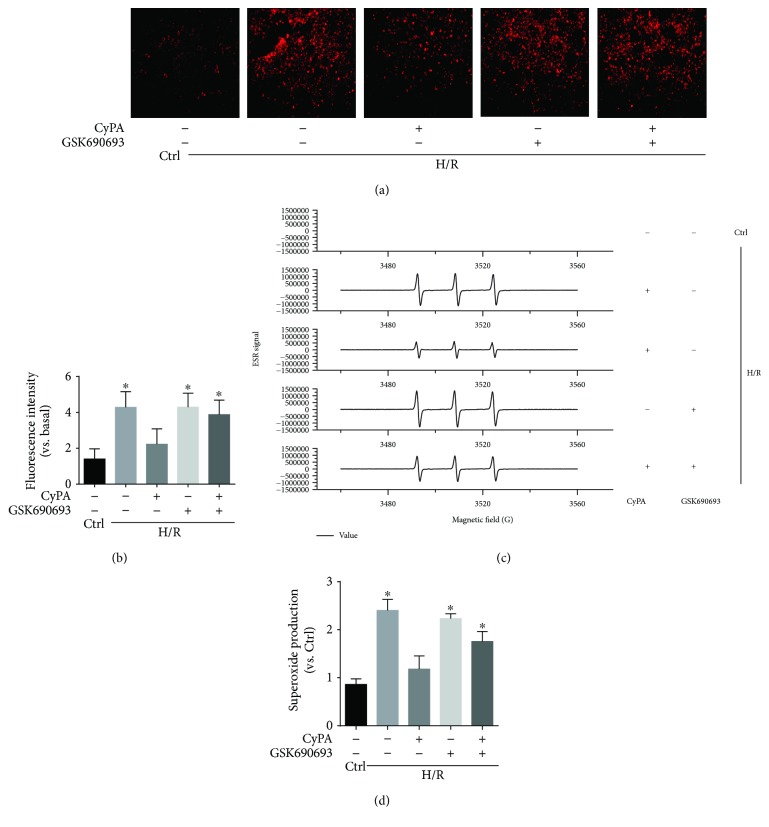
CyPA decreased O_2_^−^ production under hypoxia/reoxygenation conditions. The roles of CyPA (1000 ng/L) on O_2_^−^ production in H9c2 cells incubated under H/R conditions and the changes in the roles of CyPA (1000 ng/mL) on O_2_^−^ production after GSK690693 (10 *μ*M) intervention are revealed in representative fluorescent images for DHE staining (a) and summarized data (b). Photographs were taken at ×100 magnification. The O_2_^−^ production dependent on NADPH oxidase in the H9c2 cells is revealed in representative ESR CM nitroxide spectra resulting from CMH reacted with O_2_^−^ (c) and summarized data (d). ^∗^*p* < 0.05 vs. the H/R+CyPA group (*n* = 4).

To further explore the antioxidant effect of CyPA, we tested if CyPA treatment altered the production of O_2_^−^, a critical prooxidative stress factor. We used fluorescent spectrometry of a fluorescent probe, dihydroethidium (DHE), to detect intracellular O_2_^−^ production. H9c2 cells showed a strong red fluorescence produced from DHE oxidization by O_2_^−^ under H/R conditions. CyPA intervention obviously blocked the increase of fluorescence intensity which occurred in response to H/R. However, the H/R+GSK690693 group and the H/R+CyPA+GSK690693 group showed a strong red fluorescence (Figures 4(a) and 4(b)). By using ESR spectrometry, we further defined the role of CyPA in hypoxia/reoxygenation-induced O_2_^−^ production in H9c2 cells. Changes in ESR spectra were superoxide dismutase- (SOD-) inhibitable, as shown by the representative spectra in Figure 4(c). The summarized data in Figure 4(d) show that hypoxia/reoxygenation significantly increased O_2_^−^ production dependent on NADPH oxidase in the H9c2 cells, and this was significantly weakened by CyPA intervention, but O_2_^−^ production was significantly increased again in the H/R+GSK690693 group and the H/R+CyPA+GSK690693 group. In short, these assays showed that CyPA indeed played an antioxidant role by inhibiting NADPH oxidase-dependent O_2_^−^ production.

### 3.3. AKT/Nox2 Pathway Mediates the Role of CyPA in Opposing Hypoxia/Reoxygenation-Induced Apoptosis

**Figure 5:**
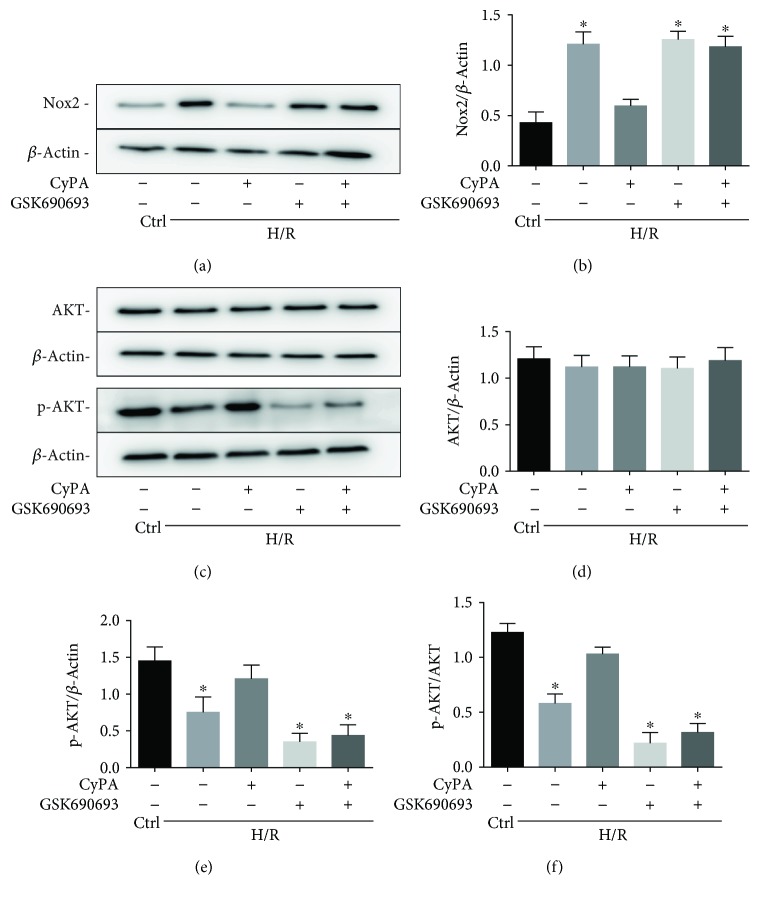
The AKT/Nox2 pathway mediates the role of CyPA in opposing hypoxia/reoxygenation-induced apoptosis, whereas inhibiting the AKT/Nox2 pathway partially abrogates CyPA-mediated protection under hypoxia/reoxygenation conditions. The expression of AKT, p-AKT, and Nox2 protein is revealed in representative western blot gel files and summarized data (a–f). ^∗^*p* < 0.05 vs. the H/R+CyPA group (*n* = 4).

**Figure 6:**
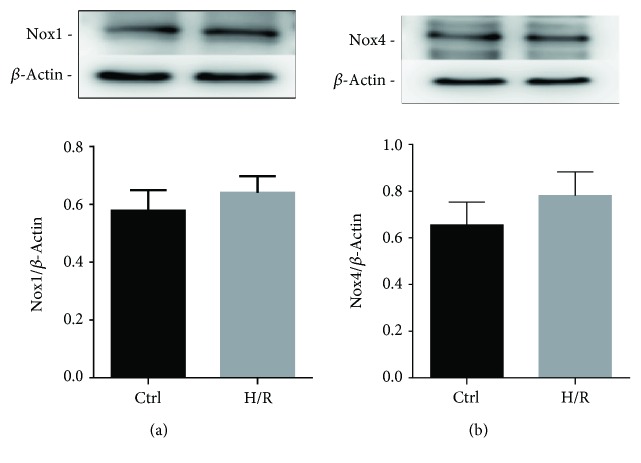
The protein expression levels of Nox1 and Nox4 are revealed in the representative western blot gel files and summarized data (a, b). ^∗^*p* < 0.05 vs. the Ctrl group (*n* = 3).

The downregulation of the production of O_2_^−^ strongly suggests involvement of the Nox2 pathway. We therefore tested the expression of Nox2 in CyPA-treated H9c2 cells by western blot analysis. As shown in Figures 5(a) and 5(b), expression of the Nox2 protein was remarkably reduced compared with the H/R group after CyPA intervention. The level of Nox2 protein is increased compared with the control group under H/R conditions (Figures 5(a) and 5(b)); to investigate the upstream pathway leading to the upregulation of Nox2 protein, we determined the activities of AKT (protein kinase B). The protein levels of p-AKT were increased significantly in response to CyPA (Figures 5(c), 5(e), and 5(f)). Although Nox2 protein has the most expression in cardiomyocytes, we have also detected other NADPH oxidases, Nox1 and Nox4, which have been shown in cardiomyocytes. We found that Nox1 protein and Nox4 protein were rarely expressed in H9c2 cells and they were not significantly increased under H/R conditions (Figure 6). These results are in agreement with the hypothesis that the AKT/Nox2 pathway mediates the role of CyPA in opposing hypoxia/reoxygenation-induced apoptosis in H9c2 cells.

### 3.4. Inhibiting the AKT/Nox2 Pathway Partially Abrogates CyPA-Mediated Protection under Hypoxia/Reoxygenation Conditions

We inhibited the AKT/Nox2 signaling pathway by using GSK690693, a pan-Akt inhibitor targeting Akt1/2/3, to determine whether there was a direct link between the effects of CyPA on AKT/Nox2 pathway activation and apoptosis. We found that the protein levels of p-AKT were significantly reduced, and the expression of Nox2 is remarkably increased, compared to the H/R+CyPA group after GSK690693 treatment (Figures 5(a)–5(f)). These results suggested that CyPA in H9c2 cells loses its antioxidant capacity under H/R conditions when the AKT/Nox2 pathway is inhibited.

### 3.5. CyPA Reduced the Expression of Nox2 Membrane-Bound Subunits to Protect Cardiomyocytes against H/R-Induced Apoptosis via the AKT/Nox2 Pathway

**Figure 7:**
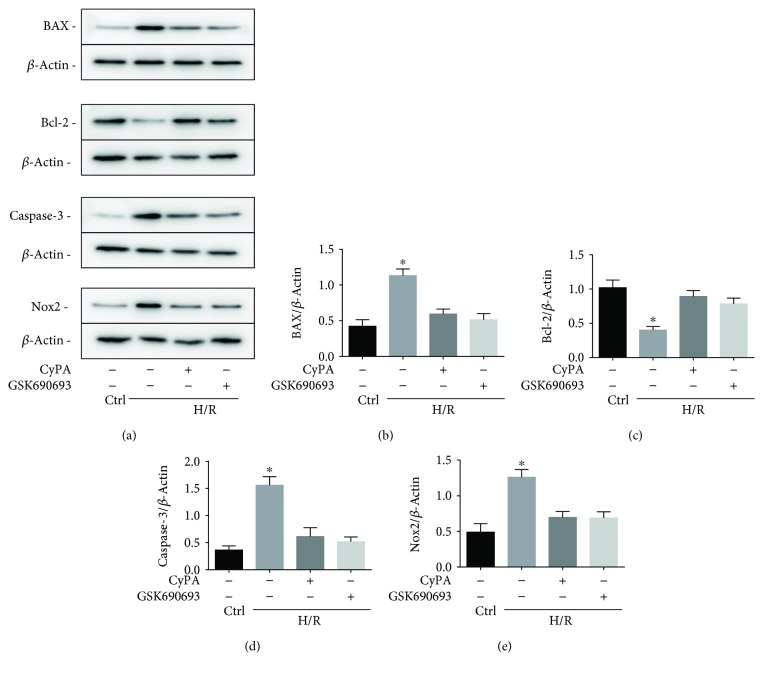
H9c2 cells were divided into four groups, control (cultured for 22 h under normoxic conditions), H/R (cultured for 18 h of hypoxia followed by 4 h of reoxygenation), H/R+CyPA (1000 ng/mL), and H/R+GSK2795039 (10 *μ*M). The protein expression levels of BAX, Bcl-2, caspase-3, and Nox2 are revealed in the representative western blot gel files and summarized data (a-e). ^∗^*p* < 0.05 vs. the H/R+CyPA group (*n* = 3).

**Figure 8:**
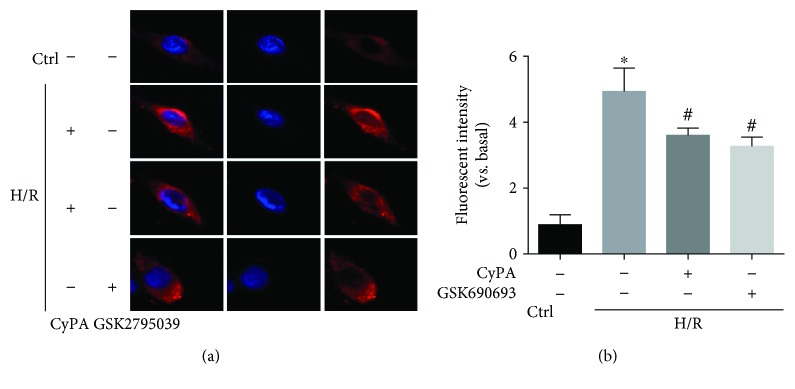
Typical representative fluorescent images showed changes of Nox2 distribution in H9c2 cells. The red-stained Nox2 moves from the cytoplasm to the plasma membrane, together with the increase of red fluorescence intensity by confocal microscopy (a) and summarized data (b). The nucleus is dyed by DAPI staining. Magnification 63x, scale bars: 10 *μ*m. ^∗^*p* < 0.05 vs. the Ctrl group (*n* = 3). ^#^*p* < 0.05 vs. the H/R group (*n* = 3).

To further demonstrate that CyPA inhibits the expression of Nox2 via the AKT/Nox2 signaling pathway in H/R conditions, we used GSK2795039 (a NADPH oxidase 2 inhibitor) to inhibit the activity of Nox2. We found that the protein levels of BAX, caspase-3, and Nox2 were remarkably reduced in the H/R+CyPA group and the H/R+GSK2795039 group (Figures 7(a), 7(b), 7(d), and 7(e)), and the expression of Bcl-2 was significantly increased in both the two groups (Figure 7(c)). In brief, CyPA inhibits the activity of Nox2 by activating the AKT signaling pathway. And we used confocal microscopy to observe the changes of Nox2 distribution in H9c2 cells. As shown in Figure 8, in normal state, Nox2 is distributed in the cytoplasm of H9c2 cells. After H/R stimulation, the expression of Nox2 on the plasma membrane increases, suggesting the role of Nox2 on apoptosis, which is modified by CyPA or GSK2795039 treatment. Regarding the present study, Nox2 appears to lead to cell apoptosis, by cytosolic subunit translocation-induced activation. These results are in agreement with the finding that CyPA reduced the expression of Nox2 membrane-bound subunits to protect cardiomyocytes against H/R-induced apoptosis via the AKT/Nox2 pathway.

### 3.6. Schematic Figure of Mechanism

**Figure 9:**
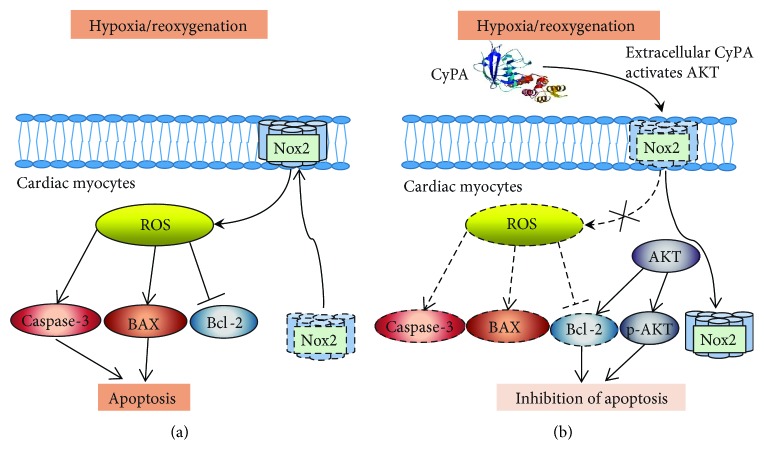
Schematic figure of mechanism.

In H/R condition, activated Nox2 can translocate to the biological membrane to increase the ROS production and then ROS upregulates the expression of caspase-3 and BAX and downregulates the expression of Bcl-2 to mediate apoptosis of cardiomyocytes (Figure 9(a)) by activating the AKT pathway. CyPA can reduce the ROS production while upregulating the expression of Bcl-2, p-AKT protein to inhibit cardiomyocyte apoptosis through this process (Figure 9(b)).

## 4. Conclusions

Until now, the role of CyPA in VSMCs and endothelial cells (ECs) has been studied in depth, but the role of CyPA in cardiomyocytes under H/R conditions has not been extensively studied. In VSMCs, CyPA promotes ROS production, and ROS further stimulates VSMC to secrete more CyPA, leading to the continuation and expansion of oxidative stress [11–13, 16, 17]. In ECs, studies have shown that CyPA can simultaneously activate the antiapoptotic and proapoptotic signaling pathways of ECs when stimulated by inflammatory factors such as ROS [23]. In addition, studies have shown that these dual effects of CyPA on ECs may be dependent on CyPA concentration [24]. In this study, we found that CyPA significantly inhibited the apoptosis of H9c2 cardiomyocytes under H/R conditions. However, CyPA in H9c2 cells lost its antioxidant capacity after using the p-AKT inhibitor. Considering that ROS mediates cellular injury in the cardiovascular system under H/R conditions [25], we believe that CyPA protects H9c2 cells against oxidative stress at least in part by inhibiting O_2_^−^ production. In order to confirm this, we examined the levels of O_2_^−^ by DHE staining. Our experimental data showed that CyPA significantly reduced O_2_^−^ production in H9c2 cardiomyocytes under H/R conditions. We also found that the production of O_2_^−^ was not reduced by CyPA stimulation in the p-AKT inhibitor GSK690693-treated group. We further confirmed this by ESR assays. One of the main sources of O_2_^−^ produced in the cardiovascular system is the NADPH oxidase enzymes [26]. The activation of these enzymes has been implicated in apoptosis. Studies have shown that Nox2 is expressed in the nucleus of cardiomyocytes during apoptosis and may be involved in proapoptotic signaling [27]. Therefore, we further tested the expression of Nox2 protein and also investigated the upstream AKT pathway and its activities. We found that CyPA in H9c2 cells mediates the activation of AKT to downregulate the expression of Nox2 protein and reduces the expression of Nox2 membrane-bound subunits, ultimately reducing the production of O_2_^−^ under H/R conditions. Through this process, CyPA can exhibit its antioxidant function. This is different from what we know about the role of CyPA in VSMCs and ECs. To rule out the effect of DMSO on cells treated with GSK690693, we examined the expression of BAX, Bcl-2, and caspase-3 in H9c2 cells treated with DMSO alone, and the results were not significantly different compared with the control group (data not shown). These results confirm our hypothesis that CyPA protects H9c2 cardiomyoblasts against H/R-induced apoptosis via the AKT/Nox2 pathway.

In previous studies on cardiovascular disease, CyPA was considered to be an inflammatory mediator, inducing apoptosis to various inflammatory cells [12, 28–30]. In addition, studies have shown that CyPA can promote cardiac hypertrophy in a mouse model [31]. However, other studies have shown that CyPA has an antioxidant effect [18–21, 32], and our experimental results demonstrate, for the first time, that CyPA has antioxidant effects on cardiomyocytes under H/R conditions. This introduces a new direction for the treatment of acute myocardial infarction and ischemia-reperfusion injury. Related clinical studies have shown that the level of CyPA in the plasma of patients with acute myocardial infarction is significantly increased [33]. On the basis of this study, we have reasons to believe that the increase of CyPA content in the plasma of patients with acute myocardial infarction may possibly protect cardiomyocytes against hypoxia/reoxygenation injury by activating the AKT/Nox2 pathway. However, due to the complex mechanisms in the different cell types, this extracellular CyPA also causes other cell injury by activating different pathways such as ERK1/2, JNK, and p38 [30]. The molecular mechanism of CyPA as an antioxidant should be explored in future studies. The ultimate goal is to use it to resist oxidative stress without causing other cardiovascular injury.

In conclusion, our data indicate that CyPA protects H9c2 cardiomyoblasts against H/R-induced apoptosis via the AKT/Nox2 pathway. The protective mechanisms of CyPA under oxidative stress give us hope that they may be a potential target for ischemia-reperfusion injury therapy.

## Data Availability

The data used to support the findings of this study are available from the corresponding author upon request.
